# Bilateral Vestibulopathy: What Can the Video Head Impulse Test Tell Us?

**DOI:** 10.3390/audiolres15020020

**Published:** 2025-02-25

**Authors:** Sofia Waissbluth, Macarena Viñuela, Emilia Escobedo, Antonia Pastore, Ivan Novoa

**Affiliations:** Department of Otolaryngology, Pontificia Universidad Católica de Chile, Santiago 8330077, Chile

**Keywords:** vertigo, dizziness, bilateral vestibulopathy, VHIT, hearing loss, oscillopsia

## Abstract

Bilateral vestibulopathy (BV) is a known cause of chronic vestibular syndrome. With the video head impulse test (VHIT), we can now evaluate all six semicircular canals independently and establish BV subgroups based on canal gain patterns. **Background/objectives**: To assess canal gain patterns for BV with VHIT, and evaluate subgroups with regard to sex, age, and hearing loss. **Methods**: A retrospective chart review was performed of all patients who underwent a VHIT between January 2021 and July 2024. Patients with decreased lateral canal gains, bilaterally, were included. Results of canal gains, VHIT patterns, audiometry, and videonystagmography (VNG) results were reviewed. **Results**: 101 cases were included. Patients were 75.5 ± 13.1 years old and 64.4% were women. Various VHIT patterns were observed; the most frequent being decreased canal gains across all six canals (44.6%), followed by a mix of canals with decreased gains with no clear pattern (34.7%). Decreased gains limited to the lateral canals were rare. We did not observe any significant difference between subgroups with regard to gender or age. Concomitant hearing loss was common (89.6%). A trend was noted, suggesting that severity of hearing loss increased with the number of affected canals. An abnormal VNG test was common (73.3%). **Conclusions**: Various patterns of canal gains were observed for patients with BV. Audiometry and VNG should be considered as part of BV studies since abnormalities are commonly found. Further research is needed to understand VHIT patterns in BV.

## 1. Introduction

Bilateral vestibulopathy (BV) is a known cause of chronic vestibular syndrome. Patients with BV present symptoms during movement, such as instability while walking or standing, blurry vision while walking, or oscillopsia, and worsening unsteadiness in the dark and/or on uneven ground, which increases the risk of falls. This condition is due to bilateral vestibular loss that can arise from end organ damage or vestibular nerve dysfunction [1]. The function of the vestibular nerve can be measured through the gains of the vestibulo-ocular reflex (VOR). The video head impulse test (VHIT) can be used to measure VOR gains for each semicircular canal separately, providing detailed information on bilateral vestibular function for high frequencies [2]. For the diagnosis of BV, it is required that the gain of both lateral semicircular canals be decreased [3]. According to the Bárány Society, the diagnosis of BV includes the presence of gains lower than 0.6 in both lateral semicircular canals with VHIT [4]. Since this test is relatively new, different patterns have been observed in the VHIT that were not previously assessable. For instance, Tarnuzer et al. described in 2016 that anterior canal function was preserved in cases of aminoglycoside vestibulotoxicity, Ménière’s disease, and BV of unknown origin, while no such sparing was found for inner-ear infections, cerebellar ataxia with neuropathy and vestibular areflexia syndrome (CANVAS), and sensorineural hearing loss [5]. The etiologies of BV are varied, with the most frequently described in the older literature being bilateral Ménière’s disease, meningitis, and ototoxicity [6]. However, more than 50% of BV cases do not have a clear diagnosis [7], and these classic pathologies above mentioned are data prior to the existence of the VHIT [8]. Therefore, there is still a wide area of research that has not been evaluated regarding BV and its findings in the VHIT.

The aim of the current study was to assess VOR gain patterns for BV with VHIT, and evaluate subgroups with regard to sex, age, and hearing loss.

## 2. Materials and Methods

A retrospective chart review was performed of all patients who underwent a VHIT between January 2021 and July 2024 at the Red de Salud UC Christus Health Center. In this period, 4170 patients underwent VHIT (removing duplicates; patients having more than one VHIT). Of all these patients, 125 presented bilateral gains in the lateral canals, less than 0.7. A total of 24 cases were eliminated because the charts were incomplete; hence, a final sample of 101 patients was reviewed. This study was approved by the local ethics committee of the Pontificia Universidad Católica de Chile, ID 241219005.

For VHIT testing, all six canals were evaluated, and the right eye was recorded (Otometrics ICS^®^ Impulse; GN Otometrics, Taastrup, Denmark). Subjects were seated, fitted with the goggles, and asked to look at an eye-level target on the wall at a 1 m distance. Following calibration, the examiner stood behind the patient and placed their hands on the participant’s head, performing the repeated head impulses. Head impulses at 150 to 300°/s were continued until 20 head impulses were adequate (i.e., artifact-free) for each tested canal. All head impulses were completed by experienced practitioners. For this study, the inclusion criterion was a lateral canal gain of <0.7 bilaterally. Although the Bárány society suggests a criterion of <0.6, we decided to expand the criteria to include all patients with 0.7 or less, as we have noticed that patients sometimes have less than 0.7 in one lateral canal and less than 0.6 in the other. Also, we do not yet fully understand the evolution of BV; hence, we decided to expand the criteria. However, we do present a subsection with data for patients that meet the Bárány criteria.

The hearing assessment was conducted using pure tone audiometry (GSI AudioStar Pro™ audiometer, Eden Prairie, MN, USA), and included air conduction thresholds obtained at 125, 250, 500, 1000, 2000, 3000, 4000, 6000, and 8000 Hz, and bone conduction thresholds at 250, 500, 1000, 2000, 3000, and 4000 Hz. A pure-tone average (PTA) > 20 dB in at least one ear was considered hearing loss. The degree of hearing loss was classified as mild (21–40 dB HL), moderate (41–60 dB HL), severe (61–80 dB HL), or profound (>80 dB HL).

We also documented the results of videonystagmography (VNG) (Interacoustics VisualEyes™ 525 system, Interacoustics; Middelfart, Denmark), which included the assessment of spontaneous nystagmus, gaze-evoked nystagmus, saccades, smooth pursuit, optokinetic testing, and positional nystagmus.

### Statistics

Descriptive statistics are presented as the mean and standard deviation for continuous variables. The Kruskal–Wallis H test with post hoc Dunn’s test for multiple comparisons was used to compare between the subgroups and the chi-square test was used for the assessment of categorical variables. A *p*-value of <0.05 was considered to be statistically significant.

## 3. Results

### 3.1. Demographics

A total of 101 patients were included, aged 75.5 ± 13.1 years old (range 29–94), and 64.4% were women. The main reason for an otolaryngology consultation and VHIT was unsteadiness (52.4%, *n* = 53), followed by episodic vestibular syndrome (31.6%, *n* = 32), acute vestibular syndrome (7.9%, *n* = 8), and chronic vestibular syndrome (7.9%, *n* = 8). Patients’ comorbidities are shown in Table 1.

### 3.2. VHIT Results

Most patients met the Bárány criteria for BV (61.4%); however, 39 patients had lateral canal gains between 0.6 and 0.69, bilaterally. Overall, the mean gains for the lateral, posterior, and anterior canals were 0.49 ± 0.16, 0.49 ± 0.18, and 0.61 ± 0.21, respectively. Of the 202 lateral canals, we observed that 72.3% also had corrective saccades. As for the posterior and anterior canals, 33.2% and 11.4% had saccades, respectively.

When reviewing the data, we observed different patterns of VOR gains for BV. We separated the patients into four subgroups based on these patterns and looked for differences regarding sex, age, and hearing loss. The subgroups were as follows:

- Group 1: decreased gains for lateral canals only (*n* = 6)
- Group 2: decreased gains in all six canals (*n* = 45)
- Group 3: decreased gains in lateral and posterior canals (*n* = 15)
- Group 4: decreased gains in lateral canals and other mixed canals (*n* = 35)

As a result, we can see that the most common presentation for VB was that all six canal gains were decreased (in 44.6% of cases), followed by a mix of canals with decreased gains with no clear pattern (in 34.7% of cases). The most infrequent VHIT presentation was seen in cases for which only the lateral canal gains were decreased (6%). Suspecting that perhaps there could be a continuum of damage throughout the vestibule and/or vestibular nerves, we assessed differences in the lateral canal gains for these four groups. While the lateral canal gains were lowest in Group 2, there was no statistically significant difference between the groups (*p* = 0.2473). We then evaluated the different gains for the vertical canals, and we observed a significant difference between the different groups, *p* = 0.0405 for the posterior canals and *p* = 0.0119 for the anterior canal gains (Table 2).

**Figure 1:**
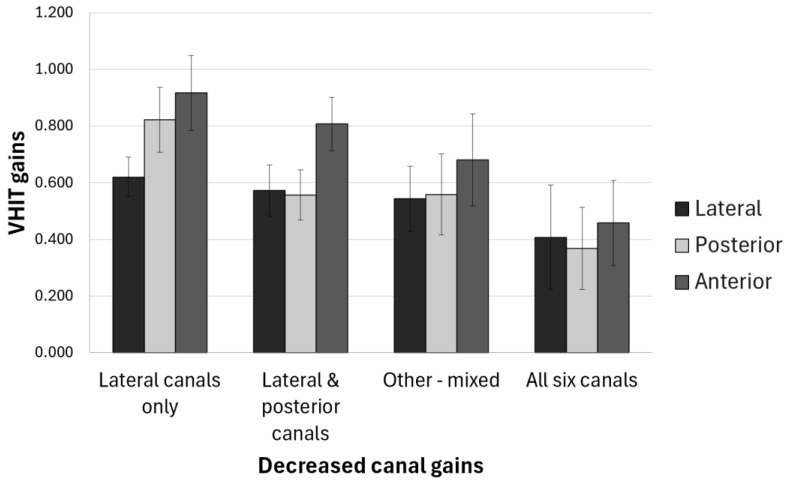
VHIT gains of all canals for patients with bilateral vestibulopathy. Mean gains ± standard deviation.

As can be seen in Figure 1, the anterior canals are the least commonly affected canals in all groups of BV, with significant differences for Group 2 with respect to all other groups (Table 3).

### 3.3. Age and Gender in Bilateral Vestibulopathy

We also evaluated whether gender or age had a relation with canal gains in BV (Table 4). When evaluating age, there was no statistically significant difference between the groups. As for gender, we can see three out of four groups had greater percentages of female gender; however, Group 1 had a small sample size. We did not observe any significant difference between groups with regard to gender.

### 3.4. Hearing Loss Assessment

We also investigated whether patients with BV experienced hearing loss and explored the relationship between the severity of hearing loss and gain patterns in VB. Of all the patients included in this study, 86.1% underwent a hearing test (pure tone audiometry). Of all these patients (*n* = 87), 78 had some degree of hearing loss (i.e., a pure tone average greater than 20 dB in at least one ear). There were no statistically significant differences between the groups with regard to having hearing loss or not (*p* = 0.8921).

**Figure 2:**
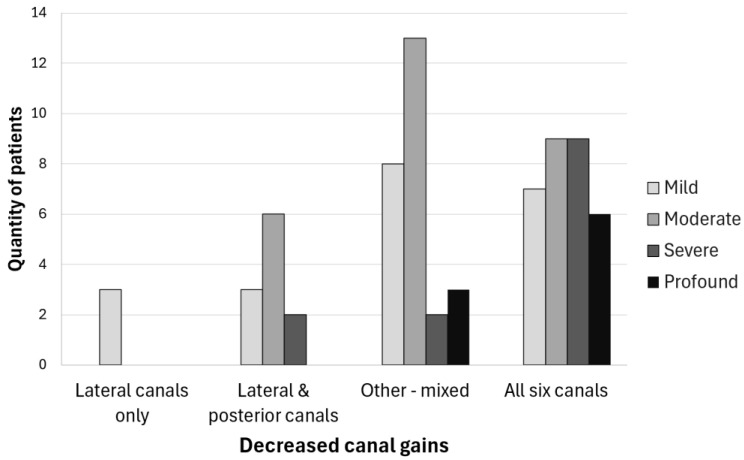
Severity of hearing loss in bilateral vestibulopathy. Degree of hearing loss: mild (21–40 dB HL), moderate (41–60 dB HL), severe (61–80 dB HL), or profound (>80 dB HL).

We then evaluated the degree of hearing loss for each subgroup (Figure 2). As we can see, only mild cases of hearing loss were found when lateral canal gains only were decreased (Group 1), and the greatest quantity of patients with profound hearing loss was found in Group 4, where canal gains were decreased for all canals. Although a trend indicating increased hearing loss severity with the number of affected canals in VB was observed, no significant association was found between the subgroup and the severity of hearing loss. Also, three patients experienced unilateral sudden sensorineural hearing loss.

### 3.5. Videonystagmography Findings

Most patients had VNG testing performed (*n* = 74, 73.3%). The main results include the abnormal saccade test (*n* = 61, 82.4%), abnormal smooth pursuit test (*n* = 50, 67.6%), abnormal optokinetic test (*n* = 36, 48.6%), spontaneous nystagmus (*n* = 29, 39.2%), positional nystagmus (*n* = 29, 39.2%), and gaze-evoked nystagmus (*n* = 11, 14.9%). In total, only twelve patients had a calorics test performed. All cases exhibited vestibular hypofunction (unilateral or bilateral), yet only one patient met the Bárány criteria for BV per calorics.

In Table 5, we can see VNG results per group. As can be observed, most patients had at least one abnormal test finding; only one patient who underwent VNG testing had a normal VNG test.

### 3.6. Potential Etiologies

Our intention was not to identify causes of VB, but to assess VHIT patterns, because this is a retrospective study, and not all patients were assessed by a neurologist or followed up at our healthcare center. In half of the cases (53.5%), a clear etiology was not determined. Potential etiologies identified were migraine (*n* = 10), vestibular migraine (*n* = 9), neurodegenerative (*n* = 5), ototoxicity (*n* = 3), vestibular neuritis (*n* = 3), vascular (*n* = 8), head trauma (*n* = 2), infectious (*n* = 2), meningitis (*n* = 1), myopathic tetraparesis (*n* = 1), genetic mitochondrial disease (*n* = 1), neoplastic (*n* = 1), and toxic (*n* = 1).

### 3.7. Bárány Criteria

Given that the Bárány Society has established lateral canal gains lower than 0.6 with VHIT as a diagnostic criterion for BV, we also chose to review our cohort using these criteria to facilitate comparisons with other studies (*n* = 65). Demographics are presented in Supplementary Table S1. The mean gains for the lateral, posterior, and anterior canals were 0.42 ± 0.16, 0.43 ± 0.17, and 0.54 ± 0.21, respectively. Gains per canal per subgroup are presented in Supplementary Table S2. Of the 130 lateral canals, we observed that 48% had corrective saccades. As for the posterior and anterior canals, 50% and 9.9% had saccades, respectively. The subgroups were as follows:

- Group 1: decreased gains for lateral canals only (*n* = 1)
- Group 2: decreased gains in all six canals (*n* = 37)
- Group 3: decreased gains in lateral and posterior canals (*n* = 15)
- Group 4: decreased gains in lateral canals and other mixed canals (*n* = 21)

The most common presentation for VB was that all six canal gains were decreased (in 56.9% of cases), followed by a mix of canals with decreased gains with no clear pattern (in 32.3% of cases). Only one case presented with decreased canal gain in the lateral canals. The anterior canals are the least commonly affected canals in all groups of BV. There was no significant difference in lateral canal gains between all subgroups (*p* = 0.6281) or vertical canals (*p* = 0.3130 for the posterior canals and *p* = 0.0710 for the anterior canal gains). An important point to note is that, due to the strict criteria, only one patient was included in Group 1; therefore, the statistics should be interpreted with caution. No patient in this cohort met the presbivestibulopathy criteria. A comparison of all subgroups across the different canals is presented in Table S3.

We did not observe any significant difference between groups with regard to age (0.9949) or gender (0.2542) (Supplementary Table S4). A total of 84.6% underwent a hearing test (pure tone audiometry). Of all these patients (*n* = 55), 49 had some degree of hearing loss. There were no statistically significant differences between the groups with regard to presenting hearing loss or not (*p* = 0.7274). One patient experienced unilateral sudden sensorineural hearing loss. There was no significant association between the subgroup and the severity of hearing loss (*p* = 0.7614).

Most patients had VNG testing performed (*n* = 50, 76.9%). The main results include the abnormal saccade test (*n* = 43, 86%), abnormal smooth pursuit test (*n* = 35, 70%), abnormal optokinetic test (*n* = 25, 50%), spontaneous nystagmus (*n* = 19, 38%), positional nystagmus (*n* = 24, 36.9%/20 cases were central and four were BPPV), and gaze-evoked nystagmus (*n* = 8, 16%). Results per subgroup are described in Table S5.

In half of the cases (50.8%, *n* = 33), a clear etiology was not determined. Potential etiologies identified were migraine (*n* = 5), vestibular migraine (*n* = 5), ototoxicity (*n* = 3), vestibular neuritis (*n* = 2), vascular (*n* = 6), head trauma (*n* = 2), infectious (*n* = 2), neurodegenerative (*n* = 3), meningitis (*n* = 1), myopathic tetraparesis (*n* = 1), genetic mitochondrial disease (*n* = 1), neoplastic (*n* = 1), and toxic (*n* = 1).

## 4. Discussion

BV is a condition characterized by reduced or absent function of both peripheral vestibular sensory organs and/or nerves. The etiologies of BV are diverse and can be categorized into several groups based on the underlying cause [9]. These include ototoxicity (i.e., aminoglycosides), neurodegenerative disorders (i.e., CANVAS, spinocerebellar ataxias), neuropathies, otoneurological disorders (Ménière’s disease, vestibular neuritis, vestibular migraine), infectious causes (i.e., meningitis, labyrinthitis, otosyphilis), autoimmune disorders (i.e., systemic lupus erythematosus, autoimmune inner ear disease), head trauma, vascular causes, genetic, and others [6,10]. While there are many causes, the precise physiopathology of BV is mostly unknown, and at least half of the cases are considered idiopathic. Migraine/vestibular migraine was the most commonly identified etiology in our cohort. The relationship between migraine and BV is not fully understood and is an area of ongoing research. However, various mechanisms have been proposed including vasospasm, cortical spreading depression, and neuroinflammation [11,12]. Our approach was to assess VHIT gain patterns in BV in order to identify subgroups and examine potential correlations with age, sex, and concomitant hearing loss.

Recently, a multicenter retrospective study of 315 patients was published. They included patients with BV; however, most of the patients were assessed with calorics or rotary chair, and only 21% of patients underwent VHIT. In this study, 56% were male patients and the mean age was 58.6 ± 15.1 years (range 7–91). Their chosen outcomes were etiology and hearing status. Interestingly, only 21% of the patients had bilateral normal hearing, and almost half had profound hearing loss in at least one ear [13]. This group of patients was younger yet presented greater hearing loss as compared to our cohort. One potential reason could be that the frequencies evaluated were different as most had reduced gains on the rotatory chair (72%) and/or bilaterally reduced caloric response (67%). It is possible that based on the potential etiology of BV, different frequencies are affected. They report that the most common etiology was idiopathic (37%), followed by a genetic cause, most frequently a COCH mutation [13]. This area of study requires further research, as genetic causes are not always diagnosable depending on the country, and may present with varying genetic profiles across different countries reporting their findings.

In another study of BV published in 2016 aiming to evaluate different etiologies and clinical subtypes, 154 patients were included with a mean age of 60 ± 12.5 years (range 19–85); 52.6% were male and the most frequent non-idiopathic etiologies were classified into genetic disorders (17%) and Ménière’s disease (16%). In this cohort, they also observed concomitant hearing loss; 84.6% had some degree of hearing loss [14]. Both aforementioned studies were completed in Europe and Asia. We had a high prevalence of hearing loss; 89.6% of patients who underwent hearing testing had some degree of hearing loss. The patients least affected by hearing loss were patients with only decreased gains in the lateral canals. We observed a trend indicating increased hearing loss severity with an increased number of affected canals in BV; however, this trend did not reach statistical significance. A recently published case-control study suggests an association between vestibular function and cognitive and motor performance, which was greater in patients with concomitant hearing loss [15]. The mechanisms linking BV, hearing loss, and cognitive performance are largely unknown.

We grouped patients into four categories based on the most common VHIT patterns; the most commonly found pattern was decreased gains in all six canals followed by decreased gains in lateral canals and other mixed canals (*n* = 35). Hence, most cases of BV will have various canals affected. The question remains whether this condition is an end-organ failure, vestibular nerve dysfunction, and/or central integration problem/degeneration. Another important finding was that most patients had at least one abnormal test on VNG, with an abnormal saccade test and abnormal smooth pursuit test being the most common findings. This raises the question as to why patients with BV commonly have central findings on VNG. Age is a crucial factor to consider, as oculomotor abnormalities can develop over time due to neuronal dropout and other age-related pathologies affecting the brain. As a result of this study, we suggest that all patients, independently of the differential diagnosis in mind, should undergo a hearing assessment as well as a VNG test.

All retrospective studies have limitations; we recognize that our study lacks knowledge of otolith function (i.e., VEMP testing), not all patients had a VNG test or brain MRI, and not all patients were evaluated by a neurologist. Also, we did not assess low-mid frequencies with calorics or rotary chair.

## 5. Conclusions

BV is a common cause of chronic vestibular syndrome. In this study, the primary reason for otolaryngology consultations and VHIT testing was unsteadiness. Most patients were older adults, although the age range spanned from 29 to 94 years, with 64.4% of patients being women. Various VHIT patterns were observed, with the most frequent finding being decreased canal gains across all six canals. Decreased gains limited to the lateral canals were rare. No significant differences were found between subgroups based on gender or age. Concomitant hearing loss was common, but no notable differences were observed between groups with or without hearing loss. A trend was noted, suggesting that the severity of hearing loss increased with the number of affected canals in BV. Most patients showed at least one abnormal finding on VNG testing, with abnormal saccades being the most common abnormality. In 53.5% of cases, no clear etiology for BV could be identified. Among those with a determined cause, the most common diagnoses were migraine/vestibular migraine and neurodegenerative disorders.

## Figures and Tables

**Table 1:** BV patient demographics.

	*n*	%
Cohort	101	100
Age	75.5 ± 13.1 years old (range 29–94)	
Sex (female)	64	64.4
High blood pressure	54	53.5
Diabetes mellitus/insulin resistance	25	24.8
Myocardial infarction/arrhythmia/cardiac insufficiency	15	14.9
Dyslipidemia	9	8.9
Transient ischemic attack/stroke	11	10.9
Traumatic brain injury	2	2
Autoimmune disorder	23	22.8
Chronic kidney disease	2	2
Migraine	10	9.9
Vestibular migraine	9	8.9
Chronic liver disease	3	3
Hearing test performed	87	86.1
Videonystagmography test performed	74	73.3
Brain MRI performed	40	39.6

**Table 2:** Mean gains of all canals for patients with bilateral vestibulopathy.

Mean Gains	Lateral Canals	Posterior Canals	Anterior Canals	Differences in Lateral Canal Gains	Differences in Posterior Canal Gains	Differences in Anterior Canal Gains
Group 1	0.62 ± 0.07	0.82 ± 0.11	0.93 ± 0.12	0.2473	**0.0405**	**0.0119**
Group 2	0.41 ± 0.18	0.36 ± 0.15	0.45 ± 0.16			
Group 3	0.57 ± 0.09	0.56 ± 0.08	0.81 ± 0.09			
Group 4	0.54 ± 0.12	0.56 ± 0.14	0.66 ± 0.19			

Group 1: decreased gains for lateral canals only (*n* = 6), Group 2: decreased gains in all six canals (*n* = 45), Group 3: decreased gains in lateral and posterior canals (*n* = 15), Group 4: decreased gains in lateral canals and other mixed canals (*n* = 35). Values in bold are statistically significant *p* values.

**Table 3:** Results of Dunn’s multiple comparison test between all groups.

*p*-Values	Group 2	Group 3	Group 4
Lateral canals			
Group 1	0.1458	0.6345	0.4496
Group 2		0.1771	0.1405
Group 3			0.8236
Posterior canals			
Group 1	**0.0126**	0.2193	0.1009
Group 2		0.0991	0.1131
Group 3			0.6667
Anterior canals			
Group 1	**0.0282**	0.5350	0.2703
Group 2		**0.0183**	**0.0223**
Group 3			0.5434

Group 1: decreased gains for lateral canals only (*n* = 6), Group 2: decreased gains in all six canals (*n* = 45), Group 3: decreased gains in lateral and posterior canals (*n* = 15), Group 4: decreased gains in lateral canals and other mixed canals (*n* = 35). Values in bold are statistically significant.

**Table 4:** Age and sex in bilateral vestibulopathy.

	Average Age (Years)	*p*-Value *	Gender (Female)	*p*-Value **
Group 1	69.3 ± 5.5	0.3696	48.6%	0.1623
Group 2	75.4 ± 15.1		73.3%	
Group 3	76.6 ± 10.3		71.1%	
Group 4	76.7 ± 12.4		66.7%	

Group 1: decreased gains for lateral canals only (*n* = 6), Group 2: decreased gains in all six canals (*n* = 45), Group 3: decreased gains in lateral and posterior canals (*n* = 15), Group 4: decreased gains in lateral canals and other mixed canals (*n* = 35). * Kruskal–Wallis H test, ** Chi-square test.

**Table 5:** Videonystagmography results in bilateral vestibulopathy.

	Saccade Test	Smooth Pursuit Test	Optokinetic Test	Spontaneous Nystagmus	Positional Nystagmus	Gaze-Evoked
Group 1 (*n* = 4)	100%	25%	50%	25%	100%	0%
Group 2 (*n* = 34)	73.5%	67.6%	41.2%	44.1%	47.1%	17.6%
Group 3 (*n* = 14)	78.6%	71.4%	35.7%	50%	78.6%	7.1%
Group 4 (*n* = 22)	95.5%	72.7%	68.2%	27.3%	63.6%	18.2%

Group 1: decreased gains for lateral canals only (*n* = 4/6), Group 2: decreased gains in all six canals (*n* = 34/45), Group 3: decreased gains in lateral and posterior canals (*n* = 14/15), Group 4: decreased gains in lateral canals and other mixed canals (*n* = 22/35).

## Data Availability

Data is unavailable due to ethical restrictions.

## Supplementary Materials

The following supporting information can be downloaded at https://www.mdpi.com/article/10.3390/audiolres15020020/s1, Table S1: BV patient demographics/Bárány criteria; Table S2: Mean gains of all canals for patients with BV/Bárány criteria; Table S3: Results of Dunn’s multiple comparison test between all subgroups/Bárány criteria; Table S4: Age and sex in bilateral vestibulopathy/Bárány criteria; Table S5: Videonystagmography results in bilateral vestibulopathy/Bárány criteria.
