# Supplementary material for: Bilateral Vestibulopathy: What Can the Video Head Impulse Test Tell Us?
Source: Audiol Res. 2025 Feb 25;15(2):20. doi: 10.3390/audiolres15020020 (PMC11932195; doi:10.3390/audiolres15020020)
Supplement: Supplementary file 1 [file audiolres-15-00020-s001.zip › audiolres-3475885-supplementary.pdf]

Supplementary file: Strict Bárány Criteria data

Table S1. BV patient demographics / Bárány criteria

	n	%
**Cohort**	65	64.3
**Age**	76.3 ± 13.4 years old<br>(range 29 - 94)	
**Sex (Female)**	42	64.6
**High blood pressure**	35	53.8
**Diabetes mellitus / insulin resistance**	14	21.5
**Myocardial infarction / Arrhythmia / cardiac insufficiency**	14	21.5
**Dyslipidemia**	7	10.8
**Transient ischemic attack / stroke**	6	9.2
**Traumatic brain injury**	2	3.1
**Autoimmune disorder**	16	24.6
**Chronic kidney disease**	2	3.1
**Chronic liver disease**	3	4.6
**Hearing test performed**	55	84.6
**Videonystagmography test performed**	50	76.9
**Brain MRI performed**	30	46.1

Table S2. Mean gains of all canals for patients with BV / Bárány criteria

<table>
<tr><th>Mean gains</th><th>Lateral canals</th><th>Posterior canals</th><th>Anterior canals</th><th>Differences in lateral canal gains</th><th>Differences in posterior canal gains</th><th>Differences in anterior canal gains</th></tr>
<tr><td>Group 1</td><td>0.49 ± 0.11</td><td>0.72 ± 0.01</td><td>0.83 ± 0.02</td><td rowspan="4">0.6281</td><td rowspan="4">0.3130</td><td rowspan="4">0.0710</td></tr>
<tr><td>Group 2</td><td>0.36 ± 0.16</td><td>0.35 ± 0.14</td><td>0.44 ± 0.15</td></tr>
<tr><td>Group 3</td><td>0.51 ± 0.09</td><td>0.55 ± 0.11</td><td>0.81 ± 0.07</td></tr>
<tr><td>Group 4</td><td>0.50 ± 0.11</td><td>0.54 ± 0.13</td><td>0.65 ± 0.18</td></tr>
</table>

Group 1: Decreased gains for lateral canals only (n = 1), Group 2: Decreased gains in all six canals (n = 37), Group 3: Decreased gains in lateral and posterior canals (n = 6), Group 4: Decreased gains in lateral canals and other mixed canals (n = 21). Values in bold are statistically significant.

Table S3. Results of Dunn's multiple comparison test between all subgroups / Bárány criteria

P-values	Group 2	Group 3	Group 4
**Lateral canals**			
Group 1	0.7230	0.8577	0.9962
Group 2		0.7069	0.1948
Group 3			0.6834
**Posterior canals**			
Group 1	0.3033	0.6536	0.5134
Group 2		0.2048	0.1726
Group 3			0.6899
**Anterior canals**			
Group 1	0.3067	0.8533	0.6492
Group 2		0.0578	**0.0377**
Group 3			0.5657

Group 1: Decreased gains for lateral canals only (n = 1), Group 2: Decreased gains in all six canals (n = 37), Group 3: Decreased gains in lateral and posterior canals (n = 6), Group 4: Decreased gains in lateral canals and other mixed canals (n = 21). Values in bold are statistically significant.

Table S4. Age and sex in bilateral vestibulopathy / Bárány criteria

	**Average age (years)**	***P*-value***	**Gender (female)**	***P*-value****
Group 1	79	0.9949	0%	0.2542
Group 2	76 ± 14.1		70.3%	
Group 3	77.5 ± 10.4		83.3%	
Group 4	76.3 ± 13.7		52.4%	

Group 1: Decreased gains for lateral canals only (n = 1), Group 2: Decreased gains in all six canals (n = 37), Group 3: Decreased gains in lateral and posterior canals (n = 6), Group 4: Decreased gains in lateral canals and other mixed canals (n = 21). *Kruskal-Wallis H test, **Chi-square test.

Table S5. Videonystagmography results in bilateral vestibulopathy / Bárány criteria

	**Saccade test**	**Smooth pursuit test**	**Optokinetic test**	**Spontaneous nystagmus**	**Positional nystagmus**	**Gaze-evoked**
Group 1 (n = 1)	100%	100%	100%	100%	0%	0%
Group 2 (n = 37)	78.6%	67.9%	42.9%	35.7%	46.4%	14.3%
Group 3 (n = 6)	66.7%	83.3%	16.7%	66.7%	100%	16.7%
Group 4 (n = 21)	100%	62.5%	68.8%	25%	56.3%	18.8%
